# Sulphuric Acid in the Treatment of Boils

**Published:** 1876-01

**Authors:** M. Marsh


Sulphuric Acid in the Treatment of Boils.—*By Dr. M. Marsh.* Dr. Marsh has used sulphuric acid, which he regards as almost a specific for boils, with constant success for twenty-five years. "As soon as the patient applies for relief, I put an adult on elixir vitriol, 20 drops three times a day, in a glass of sweetened water, one hour before meals, previously smearing the teeth well with fresh butter, in order to protect them. This is much better than sucking through a quill, and is a perfect protection, if the teeth are subsequently washed with a solution of bicarb. sod., a heaped teaspoonful to a glass of water. In the use of sulphuric acid the boil will soon melt away and disappear, no more to reappear. The acid should be kept up in ten-drop doses for at least two weeks after the boils have disappeared. To afford relief from pain and soreness, I apply a piece of common adhesive plaster, cut round, sufficiently large to cover the tumor, clipping the edges so that it will set smooth."—*Practitioner.*

